# Nosiheptide Harbors Potent *In Vitro* and Intracellular Inhbitory Activities against Mycobacterium tuberculosis

**DOI:** 10.1128/spectrum.01444-22

**Published:** 2022-10-12

**Authors:** Xia Yu, Rui Zhu, Zhi Geng, Yaoyao Kong, Fen Wang, Lingling Dong, Liping Zhao, Yi Xue, Xiaochi Ma, Hairong Huang

**Affiliations:** a National Clinical Laboratory on Tuberculosis, Beijing Key Laboratory for Drug-Resistant Tuberculosis Research, Beijing Chest Hospital, Capital Medical University, Beijing, China; b Beijing Synchrotron Radiation Facility, Institute of High Energy Physics, Chinese Academy of Sciences, Beijing, China; c Pharmaceutical Research Center, Second Affiliated Hospital, Dalian Medical University, Dalian, China; Francis Crick Institute

**Keywords:** *Mycobacterium tuberculosis*, nosiheptide, antimicrobial activity, intracellular activity

## Abstract

Multidrug-resistant tuberculosis (MDR-TB) is often associated with poor clinical outcomes. In this study, we evaluated the potential of nosiheptide (NOS) as a new drug candidate for treating Mycobacterium tuberculosis infections, including MDR-TB. The antimicrobial susceptibility testing was performed to determine the MICs of NOS against 18 reference strains of slowly growing mycobacteria (SGM) and 128 clinical isolates of M. tuberculosis. The postantibiotic effects (PAE) and interaction with other antituberculosis drugs of NOS were also evaluated using M. tuberculosis H37Rv. Fifteen out of the 18 tested reference strains of SGM had MICs far below 1 μg/mL. From the 128 M. tuberculosis clinical isolates, the MIC_50_ and MIC_90_ were 0.25 μg/mL and 1 μg/mL, respectively; the tentative epidemiological cutoff (ECOFF) was defined at 1 μg/mL. Furthermore, a Lys89Thr mutation was found in one M. tuberculosis isolate with a MIC of NOS >8 μg/mL. After 24 h of incubation, NOS at 1 μg/mL inhibited 25.79 ± 1.22% of intracellular bacterial growth, which was comparable with the inhibitory rate of 25.71 ± 3.67% achieved by rifampin at 2 μg/mL. Compared to rifampicin and isoniazid (INH), NOS had a much longer PAE, i.e., a value of about 16 days. In addition, a partial synergy between NOS and INH was observed. NOS has potent inhibitory activities against M. tuberculosis *in vitro* as well as in macrophages. Furthermore, the long PAE and partial synergistic effect with INH, in addition to the added safety of long-term use as a feed additive in husbandry, provide support for NOS being a promising drug candidate for tuberculosis treatment.

**IMPORTANCE** This study is aimed at chemotherapy for MDR-TB, mainly to explore the anti-TB activity of the existing chemotherapeutic reagent. We found that NOS has potent inhibitory activities against M. tuberculosis *in vitro* regardless of the drug-resistant profile. Furthermore, NOS also showed the long PAE and partial synergistic effect with INH and is nontoxic, providing support for its promise as a drug candidate for drug-resistant tuberculosis treatment.

## INTRODUCTION

Tuberculosis (TB) is a big challenge to public health, and 10 million new cases were estimated in 2020 worldwide by World Health Organization (WHO) (1). Multidrug-resistant tuberculosis (MDR-TB), defined as resistance to isoniazid (INH) and rifampin (RIF) simultaneously, is increasing worldwide. MDR-TB is often associated with poor clinical outcomes (2–4). However, very limited new drugs (i.e., bedaquiline, delamanid, and pretomanid) have been developed for the treatment of drug-resistant TB in the past 50 years (5, 6), which underlines the difficulties in developing new anti-TB drugs. Therefore, exploring the anti-TB activity of the existing chemotherapeutic reagent is a more efficient and time-saving strategy for improving anti-TB drug development.

Nosiheptide (NOS) is a sulfur-containing polypeptide and belongs to a family of thiazole antibiotics, which was initially isolated from Streptomyces actuosus (7). The antibacterial mechanism of NOS is inhibiting protein synthesis by binding between ribosomal proteins L11 (rplK) and 23S rRNA on the 50S subunit of the ribosome and inhibiting the GTP hydrolysis catalyzed by elongation factor EF-G (8–10). The mechanism of this action is unique and distinct from those of all the other current chemotherapeutics targeting the bacterial ribosome. Therefore, cross-resistance between them would be less expected. NOS is proven to have inhibitory activities against several Gram-positive bacteria and is considered a promising drug candidate (11). Furthermore, a previous study (12) also showed that NOS could significantly inhibit the growth of Mycobacterium avium complex (a common pathogenic slowly growing mycobacteria [SGM]) *in vitro* and in the silkworm model.

To better understand the capacity of NOS against Mycobacterium tuberculosis and other SGM, we determined its MICs against 18 SGM reference strains and 128 clinical isolates of M. tuberculosis collected in Beijing, China. We investigated the sequences of the reported NOS-resistant genes of *rplK* and 23S rRNA among the clinical isolates to identify their relationships with NOS resistance. The bactericidal activity against M. tuberculosis in macrophages was also analyzed. Furthermore, the postantibiotic effect (PAE) of NOS and interactions between NOS and other main anti-TB drugs were also characterized.

## RESULTS

### MICs of NOS against the reference strains.

The MICs of the 18 SGM reference strains for NOS are presented in Table 1. NOS demonstrated consistently strong antibacterial activity against 83.33% (15/18) of all the tested SGM species, with MICs far below 1 μg/mL, except for Mycobacterium asiaticum (MIC = 2 μg/mL), Mycobacterium marinum, and Mycobacterium nonchromogenicum (MIC >8 μg/mL).

**TABLE 1:** MICs of NOS against reference strains of 18 SGM species

Strain by Mycobacterium type	Species (strain)	MIC (μg/mL) by antimicrobial agent Nosiheptide
SGM		
ATCC 25276	Mycobacterium asiaticum	2
ATCC 25291	Mycobacterium avium	0.125
DSM 44243	Mycobacterium celatum	0.031
DSM 44622	Mycobacterium chimaera	0.5
ATCC 15754	Mycobacterium gastri	0.03
ATCC 14470	Mycobacterium gordonae	0.008
ATCC 13950	Mycobacterium intracellulare	0.25
ATCC 12478	Mycobacterium kansasii	0.125
ATCC 927	Mycobacterium marinum	>8
ATCC 19422	Mycobacterium microti	0.031
ATCC 19530	Mycobacterium nonchromogenicum	>8
DSM 44648	Mycobacterium parascrofulaceum	0.25
ATCC 27024	Mycobacterium rhodesiae	0.125
ATCC 19981	Mycobacterium scrofulaceum	0.25
ATCC 35799	Mycobacterium szulgai	0.063
ATCC 23292	Mycobacterium triviale	0.25
ATCC 27294	Mycobacterium tuberculosis *(H37Rv)*	0.125
ATCC 19250	Mycobacterium xenopi	0.008

### MICs of NOS against M. tuberculosis isolates.

A total of 128 clinical M. tuberculosis isolates, including 84 MDR-TB strains and 44 non-MDR-TB, were randomly selected to determine *in vitro* susceptibility. Among the MDR strains, 35 strains were defined as “simple MDR-TB” with susceptibility to fluoroquinolone or three second-line injectable drugs (capreomycin, kanamycin, and amikacin), whereas the other 49 strains were “MDR-TB plus,” which had additional resistance to any fluoroquinolone and at least one of three second-line injectable drugs.

NOS exhibited potent activity against all the tested M. tuberculosis isolates, including MDR-TB strains; the MICs of NOS against M. tuberculosis isolates are summarized in Table 2. Overall, most of the isolates tested (96.09%, 123/128) had MICs ≤1 μg/mL; the MIC_50_ and MIC_90_ of the M. tuberculosis isolates were 0.125 μg/mL and 1 μg/mL, respectively.

**TABLE 2:** Distribution of NOS MICs among M. tuberculosis isolates^*a*^

Classification	No. (%) of strains with MIC (μg/mL)												MIC_50_	MIC_90_
	0.016	0.031	0.062	0.125	0.25	0.5	1	2	4	8	>8	Total		
Non-MDR	0	2	6	12	5	12	5	0	1	1	0	44	0.125	1
MDR	2	8	12	26	18	10	5	1	1	0	1	84	0.125	0.5
Simple MDR-TB	0	5	9	14	5	0	1	0	0	0	1	35	0.125	0.25
MDR-TB plus	2	3	3	12	13	10	4	1	1	0	0	49	0.25	1
Total	2	10	18	38	23	22	10	1	2	1	1	128	0.125	1

a MIC_50_, concentration required to inhibit the growth of 50% of the isolates tested. MIC_90_, concentration required to inhibit the growth of 90% of the isolates tested; Simple MDR-TB, MDR-TB with susceptible to fluoroquinolone or three second-line injectable drugs (capreomycin, kanamycin, and amikacin); MDR-TB plus, MDR-TB with additional resistance to any fluoroquinolone and to at least one of three second-line injectable drugs.

Against MDR-TB isolates, the MIC_90_ of NOS (0.5 μg/mL) was lower than non-MDR isolates (1.0 μg/mL). Among the tested MDR-TB strains, the MIC_90_ of NOS in the simple MDR-TB group (0.25 μg/mL) was lower than the MDR-TB plus group (1.0 μg/mL). According to the distribution of MICs against NOS, we proposed a tentative ECOFF at 1 μg/mL to define a NOS-resistant isolate.

### Mutations conferring NOS resistance and protein alignment.

**FIG 1:**
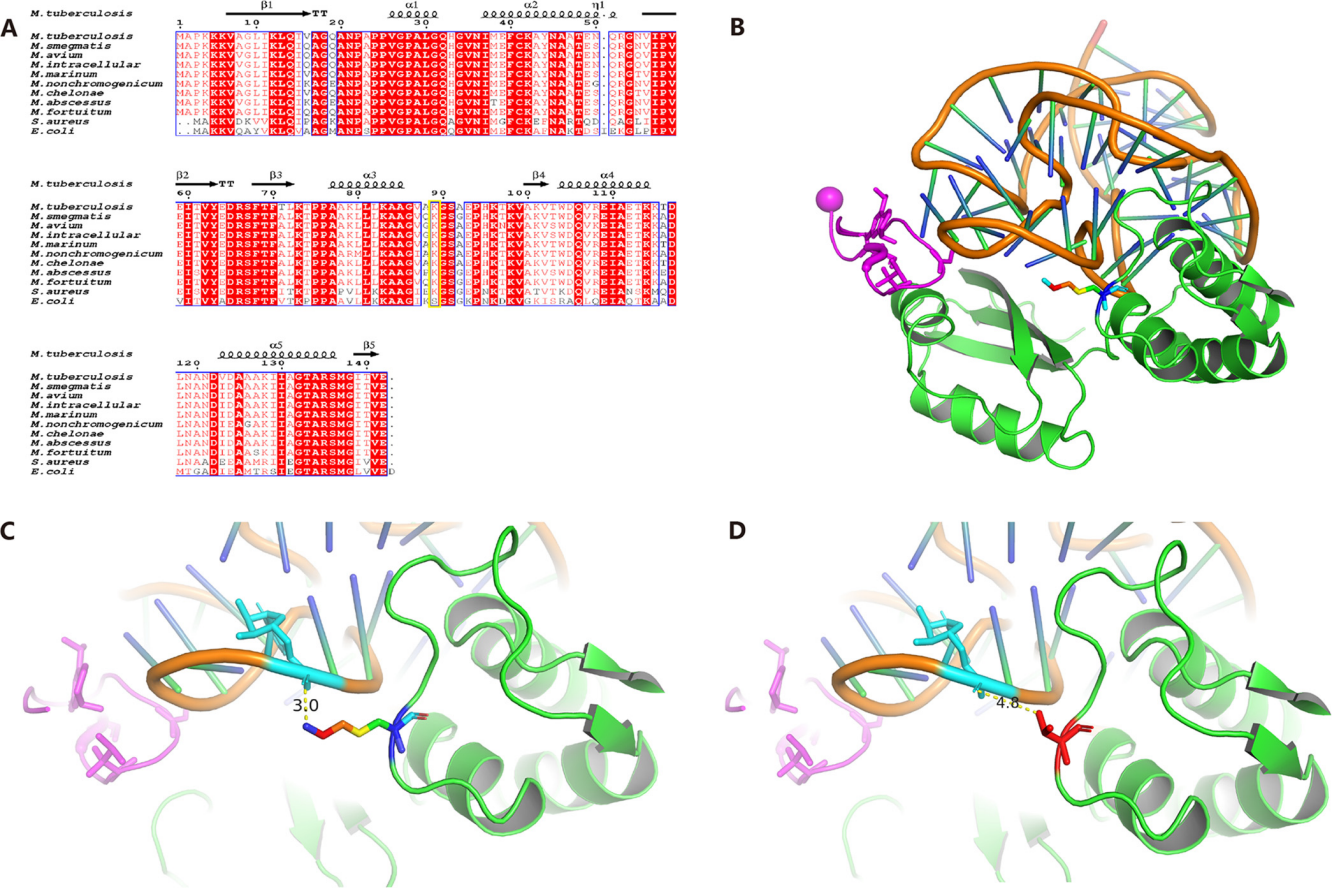
Sequence and structural alignment of *rplK homolog* proteins. (A) Alignment of the amino acid sequences of rplK in M. tuberculosis, M. smegmatis, M. avium, M. intracellulare, M. marinum, M. nonchromogenicum, M. chelonae, M. abscessus, M. fortuitum, E. coli, and S. aureus. The topology of the *rplK* encoded protein of M. tuberculosis is shown at the top. Red boxes with white letters indicate a single, fully conserved residue. The mutation in rplK of M. tuberculosis was highlighted in yellow box. (B) Structure of thiostrepton antibiotic binding to rplK in M. tuberculosis from 23S rRNA. rplK in Green and Lys89 highlighted in rainbow stick; thiostrepton antibiotic in magenta; 23SrRNA in orange and blue green gradient; (C) M. tuberculosis rplK Lys89 interacts with U1060 of 23S rRNA by hydrogen bond. (D) M. tuberculosis rplK Lys89Thr disrupts the interaction with 23S rRNA.

Multiple amino acid alignments for the *rplK* homologs of different bacterial species and the topology of proteins are shown in Fig. 1A. The protein sequences of *rplK* in different mycobacterial species were highly conserved. Except for Escherichia coli with Ser89, the amino acid at position 89 of the homologous proteins of rplK in the tested bacterium were all lysine. To determine the relationship between mutations in *rplK* homologous genes and NOS resistance, full-length *rplK* homologs of M. tuberculosis were sequenced. According to the tentative ECOFF of NOS, MIC ≥2 μg/mL was defined as resistance to NOS. Therefore, five isolates acquired MIC ≥2 μg/mL. A Lys89Thr mutation was identified in an MDR isolate with MIC >8 μg/mL, whereas no single nucleotide polymorphisms (SNPs) were detected in the remaining 127 clinical isolates. M. tuberculosis rplK Lys89 located in the loop was not adjacent to the NOS analog, while it was contacting U1060 of 23S rRNA (Fig. 1B). Notably, hydrogen bonds strengthened the intermolecular interaction between M. tuberculosis rplK Lys89 and RNA (U1060) and disrupted by rplK mutation Lys89Thr (Fig. 1C and D). In addition, no mutation was detected in the NOS binding region of the 23S rRNA.

### Bactericidal or bacteriostatic activity of NOS *in vitro*.

**FIG 2:**
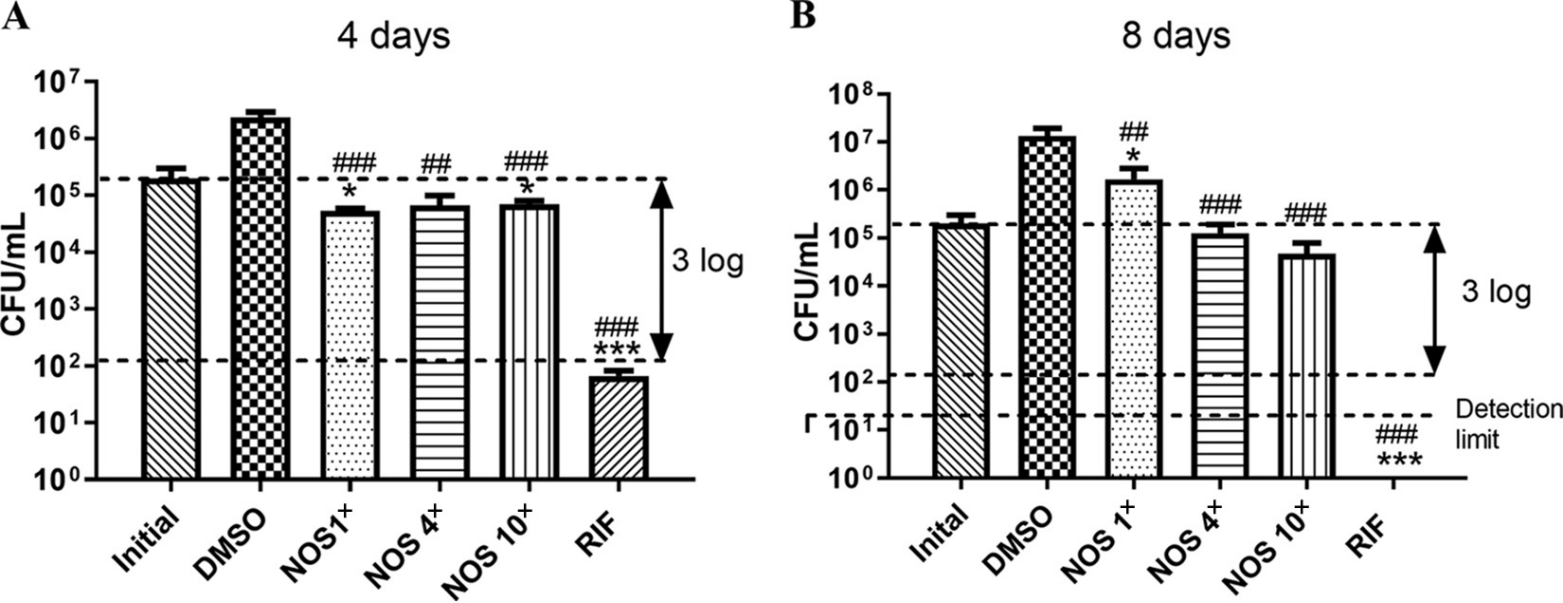
Logarithmically growing M. tuberculosis cells were exposed for 4 and 8 days to NOS at 1×, 4×, and 10× MIC values. Rifampicin (RIF) and dimethyl sulfoxide (DMSO) were used as positive and negative controls. Data were generated from three independent experiments and are shown as mean ± SD (*, *P < *0.05, ***, *P < *0.001 versus initial control; ^##^, *P < *0.01, ^###^, *P < *0.001 versus DMSO control by unpaired *t* test).

NOS displayed a MIC of 0.125 μg/mL against M. tuberculosis H37Rv and significantly inhibited bacterial growth at the concentration of 1× MIC after 4 days of incubation compared with the initial bacterial load. Except for the bacterial load that increased at 1× MIC after 8 days of incubation, the remaining conditions treated with NOS showed slight inhibitory activity compared to the initial group, and there was no statistical significance. The main reason for the regrowth may have been caused by the decay of NOS at 37°C, as the concentration of NOS in 1× MIC group after 8 days of incubation was decreased from 0.125 μg/mL to 0.012 μg/mL (Fig. S1). Compared to the DMSO group, the NOS-treated groups all presented significantly inhibited bacterial growth, whether the time of incubation was 4 or 8 days. Because NOS could not decrease the bacterial load by 3 log even at the concentration of 10×MIC, it seemed to display bacteriostatic activity *in vitro* at moderate and high concentrations (Fig. 2).

### Intracellular killing and concentration-kill assay.

**FIG 3:**
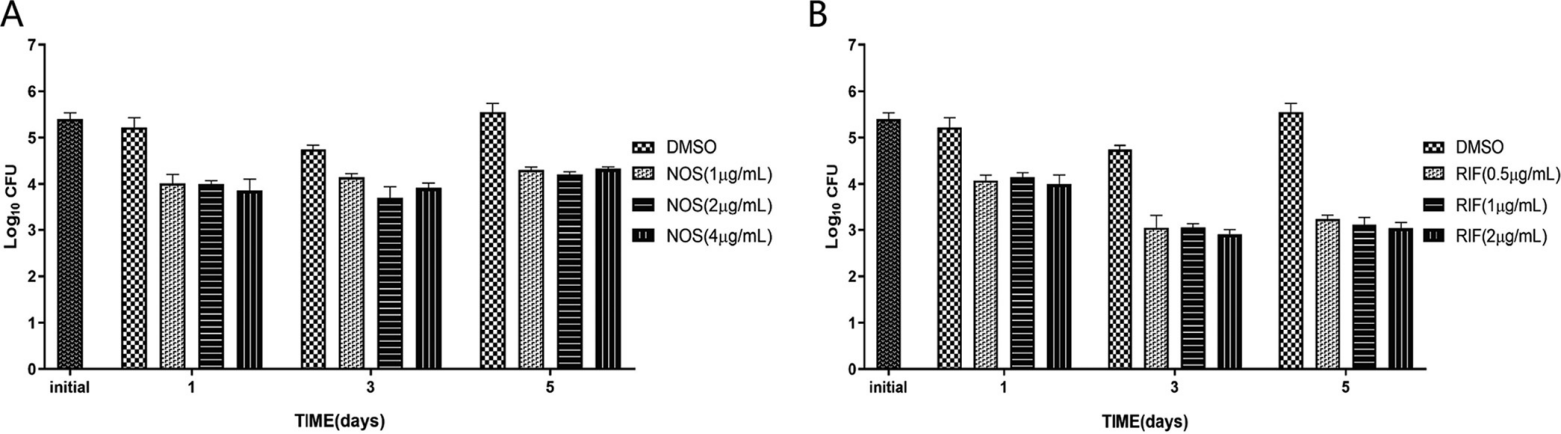
Intracellular bactericidal activities of NOS and RIF at different concentrations against M. tuberculosis in macrophages at MOI = 1:1. (A) NOS. (B) RIF. All data are shown as the means ± SD (*n* = 5).

Intracellular killing of NOS was presented in Fig. 3. At multiplicity of infection (MOI) = 1.1, reductions in colony-forming units (CFU) number were observed when treated with NOS compared to the initial control. After 24 h of incubation, NOS at 1 μg/mL, 2 μg/mL, and 4 μg/mL inhibited 25.65 ± 3.64%, 25.79 ± 1.22%, and 28.45 ± 4.64% of intracellular bacterial growth, which was comparable with that of RIF at 2 μg/mL with the inhibitory rate of 25.71 ± 1.22%. The inhibitory effects were slightly increased at day 3 postinfection, and the inhibitory rates of NOS at 2 μg/mL and 4 μg/mL were 27.95 ± 1.21% and 31.34 ± 4.42%, respectively. Compared to day 3, the bacterium presented regrowth on day 5 postinfection in all NOS-treated groups. In contrast, RIF demonstrated more robust inhibitory activities on both day 3 and day 5 postinfection (Fig. 3).

### PAE of NOS against M. tuberculosis.

**FIG 4:**
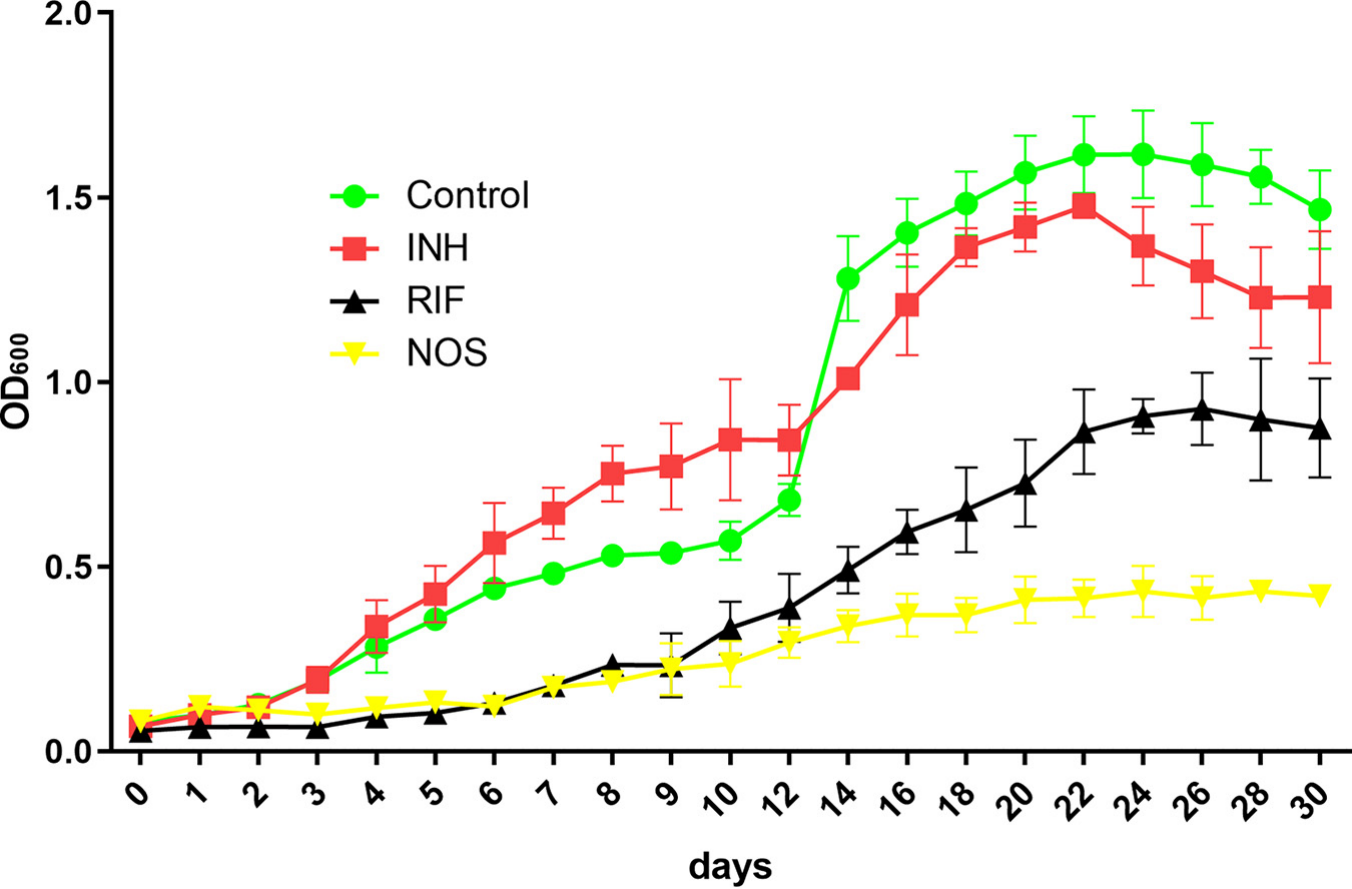
Postantibiotic effect (PAE) in the growth of M. tuberculosis H37Rv after pulse dosing with NOS, RIF, and INH.

Following 2 h of pulse exposure to 10 μg/mL of NOS and RIF, the growth of M. tuberculosis was retarded, as reflected by the long recovery time. The PAE value of NOS was found to be more than 384 h (16 days), which was far superior to RIF with a PAE value of 192 h (8 days) (Fig. 4), whereas INH showed little PAE, which was comparable with the no drug, control group.

### Checkerboard assay for compound interactions.

When used with INH, NOS showed a partial synergistic effect against M. tuberculosis H37Rv with fractional inhibitory concentrations (FICI) = 0.75 (Table 3). In addition, it showed an additive effect with bedaquiline (BDQ) and linezolid (LZD) against M. tuberculosis H37Rv, with a FICI of 1. When used in combination with RIF, clofazimine (CFZ), and moxifloxacin (MFX), NOS showed indifference against M. tuberculosis H37Rv, since the FICI was 1 to 4. No antagonistic interactions were found between NOS and the other tested compounds.

**TABLE 3:** Effectiveness of NOS in combination with antituberculosis drugs against M. tuberculosis H37Rv^*a*^

Antibiotic combination	MIC (ug/ml)		FICI	Outcome
	Antibiotics alone	Combinations		
NOS + INH	0.125/0.05	0.0625/0.0125	0.75	Partial synergy
NOS + RIF	0.125/0.032	0.125/0.016	1.5	Indifference
NOS + MFX	0.125/0.0625	0.125/0.0625	2	Indifference
NOS + LZD	0.125/0.25	0.0625/0.125	1	Additive
NOS + CFZ	0.125/0.062	0.125/0.125	3	Indifference
NOS + BDQ	0.125/0.031	0.0625/0.016	1	Additive

a BDQ, bedaquiline; CFZ, clofazimine; FICI, fractional inhibitory concentration index; INH, isoniazid; LZD, linezolid; MFX, moxifloxacin; RIF, rifampicin.

## DISCUSSION

The activities of NOS against M. avium complex and M. abscessus have been reported (12, 13), which aroused our interest to evaluate the potential of NOS as an anti-TB drug. To the best of our knowledge, this is the first study that describes the antibacterial activity of NOS against M. tuberculosis. In the present study, NOS demonstrated good antimicrobial activities against both the reference and clinical strains of M. tuberculosis. The MIC_50_ and MIC_90_ of NOS against M. tuberculosis were 0.125 μg/mL and 1 μg/mL, respectively. Most importantly, NOS demonstrated good antimicrobial activities against both drug-susceptible TB and MDR-TB strains, including the MDR-TB strains with additional resistance to fluoroquinolones and second-line injectable agents. Therefore, these outcomes suggest that NOS might be a promising candidate for the treatment of TB, including MDR-TB.

Furthermore, 15 out of 18 reference strains of different SGM species acquired MICs lower than 1 μg/mL, which also supports the usage in mycobacteria inhibition. In addition, a partial synergistic effect of NOS with INH against M. tuberculosis H37Rv was found, and additive effects were observed when combined with BDQ or LZD. This drug interaction information might be important in a regimen establishment for treating MDR-TB.

Thiazole antibiotics, including NOS and thiostrepton, affect protein synthesis inhibition by binding 50S ribosomal subunit, which is composed of 5S and 23S rRNAs and 36 riboproteins (L1 through L36). Reportedly, thiazole antibiotics interact with nucleobase A1067 and A1095 (E. coli numbering) that are located at helices 43 or 44 of the 23S rRNA, which are the binding sites of the ribosomal protein L11 (rplK), and are also the target sites of given methyltransferase and these methylations can lead thiazole resistance (9, 14, 15). However, no mutation was found in the corresponding locus of 23sRNA in the five M. tuberculosis isolates with NOS MIC ≥2 μg/mL. According to the L11-thiostrepton binding model, a proline-rich helix in the L11 protein N terminus (21-PPVGPALQQH-30) interacted with thiostrepton, which was essential for the binding of thiostrepton (9). Furthermore, *in vitro*-induced resistant strains with mutants like P22S, P23L, or G25V in Bacillus megaterium could grow at a 5-fold higher drug concentration (0.061 μg/mL/50 nM) than wild-type strains (16), suggesting that the mutation of this residue confers resistance. In our study, a new mutation Lys89Thr in *rplK* was detected in one M. tuberculosis isolate with MIC >8 μg/mL (6,545 nM). In contrast, no mutation was found in NOS susceptible isolates or strains with MIC ranging 1 μg/mL to 8 μg/mL. Additionally, M. tuberculosis rplK Lys89Thr mutation could disrupt the interaction with U1060 of 23S rRNA by structural alignment. Thus, we speculated that Lys89 may play an important role in the conformation of the complex of rplK and 23S rRNA, which consequently affected the binding of thiostrepton antibiotic. Since the appropriate breakpoint to define NOS resistance is unknown, a strain with MIC greater than ECOFF could still be a sensitive strain. Therefore, only the strain with MIC >8 μg/mL that harbored a mutation in *rplk* is plausible. Even though only a single isolate was identified, more data need to be gathered to clarify the relationship between Lys89Thr mutation in *rplK* and NOS resistance. Furthermore, other mechanisms were possible for the resistance of NOS, such as efflux or methyltransferase activities, which may need further investigation.

Although NOS has not shown bactericidal activity *in vitro*, it manifested strong antibacterial activity in the intracellular bactericidal experiment. Notably, NOS (1 μg/mL) inhibited 24.72% of bacterial growth at MOI = 1 after the incubation time of 24 h, which was comparable with RIF at 2 μg/mL. As potent activity against clinical isolates of TB and strong inhibitory activity against M. tuberculosis H37Rv in macrophages were acquired, PAE of NOS was then determined. Surprisingly, our study revealed a prolonged PAE for NOS compared with RIF (16 days versus 8 days). An extended PAE can allow longer dosing intervals without loss of therapeutic efficacy, while longer intervals reduce the costs and toxicities (17). Until now, NOS has been used as a feed additive to promote growth in pigs and poultry, which suggests it should be safe for mammalian cells (18–20). In addition, NOS was noncytotoxic in the cervical carcinoma HeLa cell line when incubated for 72 h at a concentration of 128 μg/mL, which is about 1,000-fold above the MIC against M. tuberculosis (11). Similar results were found in differentiated THP-1 cell, as the IC_50_ of NOS for 48 h of exposure were about 106.9 μM (130 μg/mL) (21). The highest safe concentration was much greater than the greatest MIC of M. tuberculosis clinical strains to NOS, which favors the possibility of NOS as a new anti-TB agent for further evaluation.

The therapeutic potential of NOS against different multidrug-resistant bacterial pathogens has been noticed gradually. For instance, NOS exhibited therapeutic efficacy against M. abscessus *in vivo*-mimic silkworm infection assay (13). Similarly, NOS provided significant (*P* < 0.03) protection against mortality against methicillin-resistant Staphylococcus aureus in a mouse model of lung infection (11). Further studies are needed to evaluate its efficacy *in vivo* and elucidate its pharmacokinetic/pharmacodynamic profile.

This study is limited because all the tested M. tuberculosis isolates were collected from a single hospital, Beijing Chest Hospital, a designated clinical center on tuberculosis, which attracts tuberculosis patients all over the country. Even so, many of the isolates may be related genetically and epidemiologically, and more clinical isolates are needed to assess the *in vitro* susceptibility of NOS in settings with different epidemiological backgrounds. In addition, poor water solubility and low bioavailability were the major concerns associated with NOS, making it difficult to achieve therapeutic doses. Notably, serials of analogs for NOS were semisynthesized, and they probably could overcome the obstacles faced by NOS in the clinical application (18).

In conclusion, NOS has potent inhibitory activities against M. tuberculosis *in vitro* and macrophages. Furthermore, the long PAE and partial synergistic effect with INH, in addition to the added safety of long-term use as a feed additive in husbandry, provide support for NOS being a promising drug candidate for TB treatment. Our data provide important insights into the potential clinical applications of NOS in treating tuberculosis infections.

## MATERIALS AND METHODS

### Ethics statement.

Ethical approval was not sought, as the study only involved laboratory testing of mycobacteria without the direct involvement of human subjects.

### Reference strains and clinical isolates.

The mycobacterial reference strains, including 18 SGM reference species, were obtained either from the American Type Culture Collection (ATCC) or from the German Collection of Microorganisms (DSM). The species constitution of these reference strains is listed in Table 1. The clinical isolates of M. tuberculosis stored in the Biobank in Beijing Chest Hospital (Beijing, China) were tested to investigate their susceptibility to NOS *in vitro*. The isolates were first cultured positive on Löwenstein-Jensen (LJ) medium, and then classified as tuberculosis preliminarily with negative results using *p*-nitrobenzoic acid-containing medium (500 μg/mL). All the strains were tested with MPT64 antigen to confirm the presence of M. tuberculosis complex (Hangzhou Genesis Biodetection And Biocontrol Co., Ltd., China). A total of 128 isolates of M. tuberculosis were recruited in Beijing chest hospital from 2019 to 2020, including 44 non-multidrug-resistant strains and 84 MDR-TB strains. More than 90% (116/128) of the isolates were from northern China and may be related genetically and epidemiologically.

### MIC testing.

NOS was purchased from Targetmol (USA) and was dissolved in dimethyl sulfoxide (DMSO) with a concentration of 8 mg/mL for the stock solution. The broth microdilution method was performed according to the guidelines of the Clinical and Laboratory Standards Institute (CLSI) (22). Middlebrook 7H9 broth (Becton, Dickinson) containing 10% oleic acid-albumin-dextrose-catalase (OADC) was used for the MIC test of M. tuberculosis and 5% for the remaining SGM using Cation-adjusted Mueller-Hinton broth. The inoculum was prepared with fresh culture grown on LJ medium. The tested drug concentrations ranged from 0.008 μg/mL (6.54 nM) to 8 μg/mL (6545 nM). Briefly, M. tuberculosis and the remaining SGM were scraped from the LJ medium, homogenized, and adjusted to 1 and 0.5 McFarland standards. Then, the suspensions were diluted and inoculated into a 96-well microtiter plate to achieve the final bacterial load at 10^5^ CFU (CFU) per well. Plates were then incubated at 37°C for 7 days, expect for *M. marinum* at 30°C. A 70-μL solution containing 50 μL Tween 80 (5%) and 20 μL Alamar Blue (Bio-Rad) was added to each well and incubated for 24 h at 37°C before color development was assessed. A change from blue to pink or purple indicated bacterial growth (23). The MIC was defined as the lowest concentration of antibiotic that prevented a color change from blue to pink.

### Mutations conferring NOS resistance and protein alignment.

Sequencing of the PCR products against M. tuberculosis isolates was performed using the Sanger method with primers designed to be specific for *rplK* and partial 23S rRNA region (C800-C1350, M. tuberculosis sequence) covering thiazole antibiotics binding site. The primers were designed as follows: rplK forward primer sequence, TCAACGCCGAACAGCAGAAA; rplK reverse primer sequence, GGTGTAGAGGTTGGTGCGG; 23S rRNA forward primer sequence, CAGCGAAAGCGAGTCTGAAT; and 23S rRNA reverse primer sequence, TTGTCGCTACTCATGCCTGC. Mutations were identified according to the outcomes of the alignments against the M. tuberculosis reference strain (ATCC 27294).

The homologous genes of *rplK* in bacterium were downloaded from the NCBI, including M. smegmatis (ATCC 19420), M. tuberculosis H37Rv (ATCC 27294), M. avium (ATCC 25291), M. intracellulare (ATCC 13950), M. marinum (ATCC 927), M. nonchromogenicum (ATCC 15930), M. chelonae (ATCC 14472), M. abscessus (ATCC 19977), M. fortuitum (ATCC 6841), S. aureus (ATCC 25923), and E.coli (*ATCC 700926*). Multiple sequence alignment of the homologous proteins was performed using the Clustal Omega software. Structure-based multiple sequence alignment was performed with ESPript 3 based on the structure of *RplK* of M. tuberculosis by AlphaFold AI system from the following website: https://espript.ibcp.fr/ESPript/ESPript/. Then, the predicted rplK in M. tuberculosis was docking to the model for thiostrepton antibiotic binding to L11 substrate from 50S rRNA(PDB: 1oln) by structural alignment.

### Bactericidal or bacteriostatic activity of NOS *in vitro*.

M. tuberculosis H37Rv (ATCC 27294) grows to early logarithmic phase and adjusted to optical density at 600 nm (OD_600_ = 0.8) and diluted 1:25 with 7H9 medium containing 10% OADC. Then, the corresponding antibiotics were added to obtain 1× MIC (0.125 μg/mL), 4× MIC (0.5 μg/mL), and 10× MIC (1.25 μg/mL) of NOS and RIF (5 μg/mL). The tubes were incubated with shaking at 60 rpm/min for 8 days at 37°C. The bacteria were enumerated at defined time intervals (4 and 8 days) by plating serial dilutions on 7H10 agar plates. An antibiotic was considered bactericidal when it can reduce 3 logs compared to the initial inoculum (24).

### Intracellular killing and concentration-kill assay.

THP-1 were seeded at 2 × 10^5^ cells/well in a 24-well plate and differentiated with phorbol myristate acetate. Cells were infected with M. tuberculosis H37Rv (ATCC 27294) at an MOI of 1 (25). At 4 h postinfection, the cells were gently washed three times with prewarmed phosphate-buffered saline (PBS) to remove the extracellular bacteria. For the intracellular killing assay, RPMI complete medium containing NOS (1 μg/mL, 2 μg/mL, and 4 μg/mL) or RIF (0.5 μg/mL, 1 μg/mL, and 2 μg/mL) were added. The culture medium with DMSO was included as a growth control. At the treatment time of 1, 3, and 5 days postinfection, macrophages were extensively washed with PBS and lysed with 0.1% Trion-X. CFU were quantified by plating serial dilutions of lysates on 7H10 agar plates. The bacterial survival rate was calculated using the formula: viability = CFU of bacteria under the treatment of NOS or RIF/CFU of the initial bacterial load × 100%.

### Postantibiotic effect.

PAEs for NOS and control drugs were determined using a previously described approach with slight modifications (26). In 7H9 broth, M. tuberculosis H37Rv (ATCC 27294) in the log phase (OD_600_ = 0.6) were exposed to NOS, RIF, and INH at the same concentration of 10 μg/mL for 2 h. The exact concentration of bacterial cells with only solvent (DMSO) was used as a positive control. Only fresh medium without any bacteria and drugs was used as a negative control. Following 2 h of drug treatment at 37°C, antibiotics were removed by centrifugation (8,000 rpm, 10 min), and the cell pellets were washed three times in prewarmed fresh 7H9 broth. Finally, washed pellets were resuspended in prewarmed 7H9 broth and incubated at 37°C until they reached growth saturation (OD_max_). The OD_600_ was determined for each culture before drug exposure, after drug removal, and at 24-h intervals after that. The duration of the PAE was calculated as the time taken for the antibiotic-treated culture to reach 50% OD_max_ of the drug-free culture minus the time taken for the drug-free control to achieve the same point (26).

### FIC index determination.

A checkerboard assay evaluated drug-drug interactions to find the best combination with NOS. The interactions were determined by using alamarBlue by checkerboard assays with 2-fold serially diluted concentrations in final volumes of 200 μL (27). The checkerboard method was used to evaluate the antibacterial ability of the two antibacterial drugs. One microliter of the 2-fold serial dilutions of each test compound (starting from 8× MIC to 0.125×MIC) was prepared in a well of a 96-well microplate (98 μL per well). Bacterial stocks of M. tuberculosis from the exponential-phase cultures were performed as MIC assay and added to the plates to obtain a total volume of 100 μL. Each plate was then incubated for 7 days at 37°C before adding alamarBlue.

Six anti-TB drugs with different targets were selected, including INH (cell wall), RIF (RNA polymerase), MFX (mycobacterial DNA gyrase), LZD (23S ribosome), CFZ (outer membrane), and BDQ (ATP synthase). For evaluating compound interactions, FICs were calculated using the following formula: ΣFIC = [MIC of compound X in combination with Y]/[MIC of X alone] + [MIC of compound Y in combination with X]/[MIC of Y alone]. Synergy was defined by ΣFIC values: ≤0.5, synergy; >0.5 to 0.75, partial synergy; >0.75 to 1.0, additive effect; >1.0 to 4.0, indifference; and >4.0, antagonism (26).

### Statistical analysis.

Experiments were repeated in triplicate, with a minimum of triplicate data points per experiment. Data were analyzed using GraphPad Prism 8.0. The means of the drug-free control and drug-treated group were compared using an unpaired Student’s *t* test. The epidemiological cutoff (ECOFF) was determined according to the distribution profile of the MIC values. For the unimodal MIC distribution profile, ECOFF was defined as the concentration that could inhibit 95% of the bacterial population; For the bimodal MIC distribution profile, ECOFF was set between the two populations (28).

### Data availability.

All data relevant to this study are supplied in the manuscript and supplemental material or are available from the corresponding author upon request. Supplemental Table S1 and Fig. S1 and the methods for NOS stability monitored by HPLC are provided in the supplemental material.
