# Supplementary material for: Nosiheptide Harbors Potent In Vitro and Intracellular Inhbitory Activities against Mycobacterium tuberculosis
Source: Microbiol Spectr. 2022 Oct 12;10(6):e01444-22. doi: 10.1128/spectrum.01444-22 (PMC9769715; doi:10.1128/spectrum.01444-22)
Supplement: Supplemental file 1 — Supplemental materials and methods and Fig. S1. Download spectrum.01444-22-s0002.pdf, PDF file, 0.3 MB [file spectrum.01444-22-s0002.pdf]

Supplementary material

1. Materials and methods

1.1Nosiheptide (NOS) stability monitored by HPLC methods with fluorescence (FL) detection

1.1.1Medium preparation.

The 7H9 broth (BD, the United States) was prepared according to the manufacturer's instruction. After sterilizing, Oleic acid-albumin-dextrose-catalase (OADC) (10%; BD, the United States) was added into broth medium.

1.1.2 Standard solutions.

NOS standards (purity = 97.05%) were supplied by MedChemExpress Company (the United State). The standard solution of NOS was prepared by weighing 1 mg NOS into 1 mL Dimethyl sulfoxide (DMSO) (Sigma, Germany). This solution (1000 µg/mL) was used as a stock solution and stored at −80 ℃ for 3 months. And working solutions of appropriate concentrations were prepared as follows. Briefly, the stock solution was diluted to a series of concentrations (78.125 µg/L, 156.25 µg/L, 312.5 µg/L, 625 µg/L, 1250 µg/L, 2500 µg/L and 5000 µg/L) with 7H9 broth. The mixture was deproteinized by adding a 4-fold volume of methanol and then centrifuged at 3,500 g for 10 min, and the supernatant was collected for HPLC-FL analysis.

1.1.3 Sample preparation.

Stability was monitored by NOS at the concentrations of 0.125 µg/mL, 0.5 µg/mL and 1.25 µg/mL, i.e.,1*MIC,4*MIC and 10*MIC of NOS against *M.tuberculosis* H37Rv,respectively.Samples in triplicate were prepared by fortifying the appropriate volume of stock solution into the 7H9 broth and the kept at 37℃. The concentrations of NOS were monitored on day 2, 4 and 8.

1.1.4 Instrumentation and chromatographic conditions.

HPLC system (Agilent Technologies, the United State) was equipped with an autosampler (G1329A), Fluorescence Detector (G1321A) and column heater (G1316A). Chromatographic separation was performed via an Agilent ZORBAX

SB-C18 column (2.1 x 50 mm; Agilent Technologies, the United State) for NOS and the column was operated at 30 ℃(1). The mobile phase constituted acetonitrile-5 mM/L ammonium formate and 0.01% formic acid solution (50:50, v/v) at a flow rate of 0.2mL/min(1, 2). Fluorescence of NOS was measured by excitation at 357 nm and emission at 515 nm, respectively(3). Inject 10 μL aliquot into HPLC.

Supplementary Figure. S1

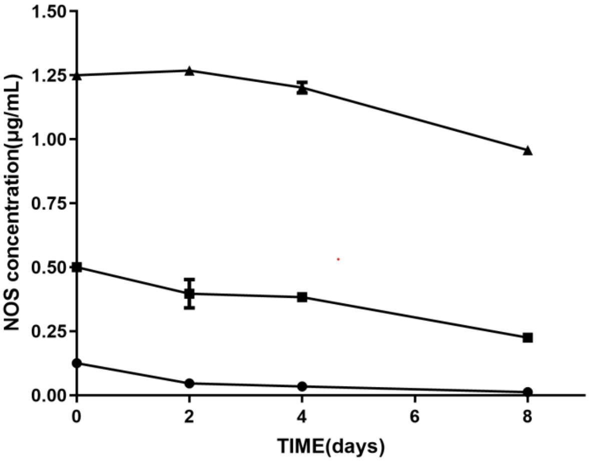

Figure S1.Serial monitoring outcomes of NOS stability in 7H9 broth when kept at 37 ℃.

References:

1. Shen J, Zhao F, Zhu P, Wu F, Chen X, Kang H, Yue Z. 2022. Direct determination of nosiheptide residue in animal tissues by liquid chromatography-tandem mass spectrometry. J Chromatogr B Analyt Technol Biomed Life Sci 1193:123167.
2. Horii S, Oku N. 2000. Rapid determination of nosiheptide in meat and egg by liquid chromatography with fluorescence detection. J AOAC Int 83:17-9.
3. Xie J, Song X, Zhang Y, Zhang M, Li X, He L. 2019. Rapid determination of nosiheptide in feed based on dispersive SPE coupled with HPLC. J Sep Sci 42:706-715.
